# Healthcare Utilization and Costs Among US Adolescents With Alopecia Areata

**DOI:** 10.36469/001c.36229

**Published:** 2022-07-29

**Authors:** Markqayne Ray, Elyse Swallow, Kavita Gandhi, Christopher Carley, Vanja Sikirica, Travis Wang, Nicolae Done, James Signorovitch, Arash Mostaghimi

**Affiliations:** 1 Pfizer Inc, Collegeville, Pennsylvania; 2 Analysis Group, Boston, Massachusetts; 3 3Brigham and Women’s Hospital, Harvard Medical School, Boston, Massachusetts

**Keywords:** alopecia areata, healthcare costs, adolescents, retrospective claims study, corticosteroids

## Abstract

**Background:** Alopecia areata (AA) is an autoimmune disease of hair loss affecting people of all ages. Alopecia totalis (AT) and universalis (AU) involve scalp and total body hair loss, respectively. AA significantly affects quality of life, but evidence on the economic burden in adolescents is limited.

**Objectives:** To assess healthcare resource utilization (HCRU) and all-cause direct healthcare costs, including out-of-pocket (OOP) costs, of US adolescents with AA.

**Methods:** IBM MarketScan® Commercial and Medicare databases were used to identify patients aged 12-17 years with ≥2 claims with AA/AT/AU diagnosis (prevalent cases), from October 1, 2015, to March 31, 2018, enrolled for ≥12 months before and after the first AA diagnosis (index). Patients were matched 1:3 to non-AA controls on index year, demographics, plan type, and Charlson Comorbidity Index. Per patient per year HCRU and costs were compared post-index.

**Results:** Patients comprised 130 AT/AU adolescents and 1105 non-AT/AU adolescents (53.8% female; mean age, 14.6 years). Post-index, AT/AU vs controls had more outpatient (14.5 vs 7.1) and dermatologist (3.6 vs 0.3) visits, higher mean plan costs (9397vs2267), including medical (7480vs1780) and pharmacy (1918vs487) costs, and higher OOP costs (2081vs751) (all *P*<.001). The non-AT/AU cohort vs controls had more outpatient (11.6 vs 8.0) and dermatologist (3.4 vs 0.4) visits, higher mean plan costs (7587vs4496), and higher OOP costs (1579vs805) (all *P*<.001).

**Discussion:** This large-sample, real-world analysis found that adolescents with prevalent AA had significantly higher HCRU and all-cause costs than matched controls. The greater burden was driven by more frequent outpatient visits, and higher payer medical and pharmacy costs in comparison with controls. Oral corticosteroid use was higher among patients with AT/AU; topical and injectable corticosteroid use was higher for non-AT/AU. Although the data preclude the identification of AA-attributable costs, the matched-control design allows an estimation of incremental all-cause costs associated with AA.

**Conclusions:** Adolescents with AA incurred substantial incremental healthcare costs, with greater costs incurred among those with AT/AU. Study findings suggest that AA incurs costs as a medical condition with a high burden on adolescent patients and health plans.

## BACKGROUND

Alopecia areata (AA) is an autoimmune skin disease characterized by nonscarring hair loss.^1^ Patients with AA can present with small patches of hair loss; complete loss of scalp hair, known as alopecia totalis (AT); or complete loss of scalp, facial, and body hair, known as alopecia universalis (AU).^1–3^ Less common forms include a band-like area of hair loss around the scalp (ophiasis), a halo-like form (ophiasis inversus [sisaipho]),^4^ and others.^5–7^

AA affects up to 147 million people worldwide and was found to have an estimated prevalence of 0.21% in the United States in 2017, with a lifetime prevalence risk as high as 2.51%.^8–11^ Although this condition can affect individuals of all ages, AA is highly prevalent among adolescents,^12^ with studies estimating that 20% of patients with AA are younger than 16 years of age.^13–15^ It is estimated that 34% to 50% of patients with AA recover within a year, without the need for treatment.^10,16–19^ However, many patients experience relapsing or remitting disease,^10,16–20^ and between 10% and 35% of patients can ultimately experience either AT or AU.^10,16–19^

The burden of AA is often exacerbated by the presence of comorbid conditions such as atopic dermatitis, lupus erythematosus, psoriasis, asthma, and allergic rhinitis,^21–23^ some of which may share similar underlying inflammatory/ autoimmune mechanisms.^24^ AA diminishes health-related quality of life in nearly half of patients and is associated with an approximately 70% lifetime prevalence of psychiatric disorders, including major depression and anxiety disorders.^25–28^ Moreover, the rate of psychiatric comorbidity is particularly high among children and adolescents with AA, with as many as 78% of adolescent patients having at least 1 lifetime psychiatric disorder.^13,29,30^ Adolescent patients may also be more vulnerable to the potential psychosocial complications arising from AA itself, stressing the utmost importance of early diagnosis and improved management.^13^ Despite these concerns, the true burden of disease among patients with AA is likely to be underestimated because of the limited body and scope of existing research.^25,31,32^

Although a wide range of topical and systemic medications are currently used to manage AA, none are approved by the US Food and Drug Administration, and effective treatments for persistent or extensive disease remain scarce.^18,33–36^ Corticosteroids, whether in topical, injectable, or oral formulations, alone or in combination, remain the most widely prescribed treatments for adolescents with acute and extensive AA.^37,38^ Additionally, calcineurin inhibitors, immunomodulators (eg, methotrexate), and Janus kinase inhibitors may be considered as options for more severe AA.^1,37,38^ However, further high-quality evidence is warranted to support choices among the available AA therapies.^33,34,37,39^

Prior research suggests that the clinical burden of AA may translate into a considerable economic burden for payers and patients.^40^ However, evidence regarding the costs and healthcare resource utilization (HCRU) associated with AA, particularly in younger patients, remains limited. In 1 retrospective claims analysis, AA patients incurred incremental total medical costs of approximately $3000 per year compared with matched controls in a combined population of adolescents and adults from a managed care population.^41^ A separate survey-based study reported that adult patients with AA incurred average out-of-pocket (OOP) expenses of approximately $1350 annually.^42^ A comprehensive analysis of the economic effect of AA among adolescents is needed to better address the unmet need in patients affected by this disease.

We analyzed HCRU and all-cause direct healthcare costs, including payer and OOP costs, in US adolescent patients with AT/AU or non-AT/AU vs non-AA matched-control groups. Medication use related to AA and its associated comorbidities was also analyzed as a secondary research objective.

## METHODS

### Data Source

This study utilized data from the IBM MarketScan® Commercial and Medicare Supplemental health insurance claims databases, which contain data from approximately 100 different insurance providers and third-party administrators in the United States. The databases include enrollment history and claims for medical (provider and institutional) and outpatient pharmacy services. Inpatient service records are available at both the claim level and summarized stay level. Data are de-identified and comply with the patient confidentiality requirements of the Health Insurance Portability and Accountability Act (HIPAA). As a result, no institutional review board approval was required. Claims and health plan coverage data were available between January 1, 2014, and March 31, 2019 (data period).

### Study Design and Patient Selection

**Figure 1.**
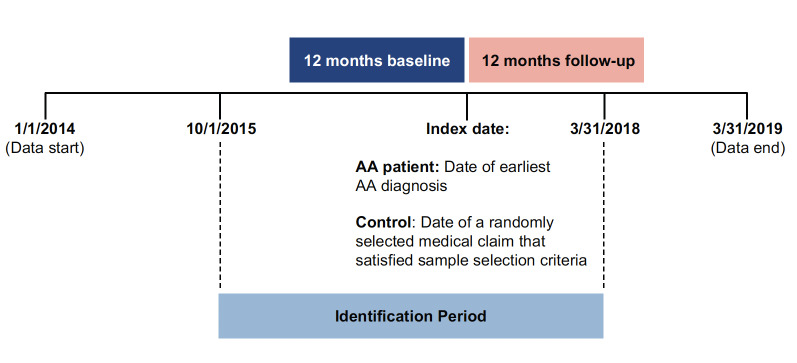
Study Design Abbreviation: AA, alopecia areata.

A retrospective cohort study was used to describe and compare economic outcomes and medication use in adolescents of 12 to 17 years of age with a diagnosis of AA and controls without AA matched on demographic, insurance, and clinical characteristics. Patients in both cohorts were required to be between 12 and 17 years of age on the index date (as defined below) and continuously enrolled in a health insurance plan for at least 12 months before the index date (defined as the baseline period) and for at least 12 months after the index date (defined as the follow-up period). Patients in the AA cohort were required to have at least 2 inpatient or outpatient claims with a diagnosis of AA (*International Classification of Diseases, Tenth Revision, Clinical Modification* [ICD-10-CM]: L63.x) from October 1, 2015, to March 31, 2018 (identification period). The ICD-10-CM codes replaced ICD-9-CM codes on October 1, 2015, and, due to their greater granularity, allow for the differentiation of 5 AA manifestations: alopecia (capitis) totalis (L63.0), alopecia universalis (L63.1), ophiasis (L63.2), other alopecia areata (L63.8), and alopecia areata, unspecified (L63.9). Eligible controls had no claims with a diagnosis code for AA (ICD-9-CM code: 704.01 or ICD-10-CM code: L63.x) throughout the data period. The ICD-9-CM codes do not distinguish between different AA manifestations and were therefore not used for identification of patients with AA. However, controls were required to have neither ICD-9-CM (before October 1, 2015) nor ICD-10-CM codes (after October 1, 2015) for AA. The index date was defined as the earliest AA diagnosis date for patients with AA and as the date of a randomly assigned medical claim for control patients (Figure 1). Controls with missing enrollment information were excluded. No diagnosis washout period was required; thus, the study sample represents prevalent AA cases.

For the subgroup analysis, eligible patients were grouped according to whether they had AA with AT or AU (AT/AU subgroup) or AA without AT or AU (non-AT/AU subgroup). Patients with AT/AU were identified by at least 1 diagnosis of AT (ICD-10-CM: L63.0) or AU (ICD-10-CM: L63.1) on the index date or at any point thereafter during the data period. Patients with non-AT/AU thus included patients with AA identified based on codes for ophiasis, other AA, or unspecified AA (ICD-10-CM L63.2-L63.9) but no AT or AU codes during the data period. Patients in both the AT/AU and non-AT/AU subgroups were separately matched 1:3 with non-AA controls on age, sex, region, index year, health insurance type, and overall Charlson Comorbidity Index (CCI) score.

### Measurements and Outcomes

Patient demographics and clinical characteristics were assessed during the 12-month baseline period and included age, sex, geographic region, insurance type, CCI score, AA-related individual comorbidities, and medications for AA (ie, topical/ injectable/oral corticosteroids) and related comorbidities (ie, antidepressants, anxiolytics). During the 12-month post-index follow-up period, study outcomes were summarized and compared between cohorts and included all-cause HCRU (ie, outpatient visits and dermatologist visits) and all-cause healthcare costs. Healthcare costs included payer costs, defined as the amount reimbursed by the commercial plan and coordination of benefits (ie, supplemental insurance and Medicare-paid amounts), and OOP payer costs, defined as copayments, deductibles, and coinsurance. As a secondary outcome of interest, medication use for AA and related comorbidities was summarized and compared between cohorts during follow-up.

### Statistical Analysis

Summary statistics were calculated for demographic and clinical characteristics at baseline and for patient outcomes during the 12-month post-index follow-up period. Means and SD were reported for continuous variables; frequencies and proportions were reported for binary and categorical variables. Mean per patient per year (PPPY) HCRU and all-cause healthcare costs were compared post-index in the AT/AU subgroup vs matched controls and separately in the non-AT/AU subgroup vs their matched-control group using 2-sample *t* tests for continuous variables and χ^2^ tests for categorical variables. Healthcare costs were inflated to 2018 US dollars using the medical care component of the Consumer Price Index. Analyses were conducted using SAS software version 9.4 (SAS Institute, Cary, North Carolina) and R version 3.6.1 (R Foundation, Vienna, Austria).

## RESULTS

### Baseline Characteristics

**Figure 2.**
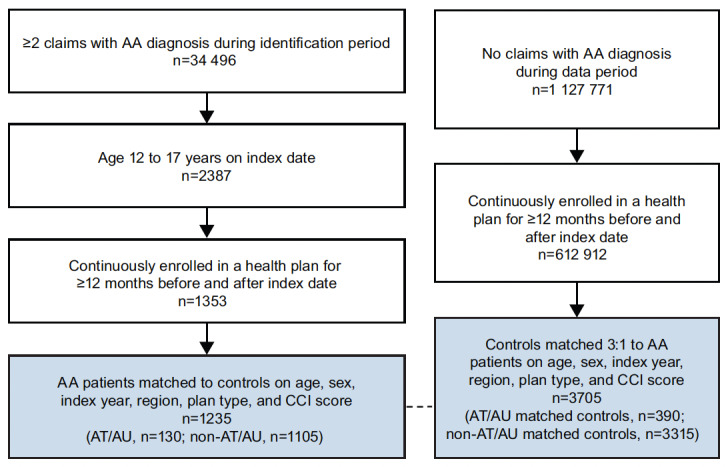
Sample Selection Abbreviations: AA, alopecia areata; AT, alopecia totalis; AU, alopecia universalis.

After applying inclusion and exclusion criteria, the study included 1235 adolescents with AA and 3705 matched unaffected controls (Figure 2). Of the 1235 patients with AA, 130 (10.5%) had the AT/AU subtype (Table 1). Most patients with non-AT/AU and AT/AU were female (53.9% and 53.1%, respectively); mean age (SD) as of the index date was 14.6 (1.6) years in both subsamples. The AA group vs matched controls had higher rates of any selected atopic diseases (non-AT/AU: 22.8% vs 17.4%, *P<*.001; AT/AU: 24.6% vs 16.7%, *P*=.059), including atopic dermatitis (non-AT/AU: 6.2% vs 2.1%, *P<*.001; AT/AU: 6.2% vs 1.3%, *P=*.006). Patients in the AA group also had higher rates of autoimmune diseases (non-AT/AU: 5.0% vs 2.2%, *P*<.001; AT/AU: 6.2% vs 2.6%, *P*=0.097) vs matched controls at baseline, including Hashimoto’s disease (non-AT/AU: 1.4% vs 0.6%, *P*=.034; AT/AU: 3.1% vs 0.8%, *P*=.069), type 1 diabetes mellitus (non-AT/AU: 1.4% vs 0.7%, *P*=0.044; AT/AU: 0.8% vs 1.3%, *P*=1.000), psoriasis (non-AT/AU: 1.2% vs 0.4%, *P*=.006; AT/AU: 0.8% vs 0.0%, *P*=.250), and vitiligo (non-AT/AU: 0.7% vs 0.2%, *P=*.006; AT/AU: 0.8% vs 0.3%, *P*=0.438). As a consequence of matching, mean CCI score was balanced at 0.1 in both cohorts of the non-AT/AU and AT/AU subgroups; among non-AT/AU and AT/AU subgroups and matched controls, 9.4% and 11.5% had a CCI score of 1 or greater, respectively.

**Table 1.** Baseline Patient Demographics and Comorbidities

	**Patients With Non-AT/AU**			**Patients With AT/AU**		
**Characteristic**	**Non-AT/AU (n=1105)**	**Matched Controls Without AA (n=3315)**	***P* Value**	**AT/AU (n=130)**	**Matched Controls Without AA (n=390)**	***P* Value**
Age (years), mean ± SD	14.6±1.6	14.6±1.6	1.000	14.6±1.7	14.6±1.7	1.000
Sex, n (%)			1.000			1.000
Female	596 (53.9)	1788 (53.9)		69 (53.1)	207 (53.1)	
Male	509 (46.1)	1527 (46.1)		61 (46.9)	183 (46.9)	
US region, n (%)			1.000			1.000
South	431 (39.0)	1293 (39.0)		52 (40.0)	156 (40.0)	
Northeast	283 (25.6)	849 (25.6)		35 (26.9)	105 (26.9)	
Midwest	239 (21.6)	717 (21.6)		19 (14.6)	57 (14.6)	
West	152 (13.8)	456 (13.8)		24 (18.5)	72 (18.5)	
Insurance type, n (%)			1.000			1.000
Managed care^a^	830 (75.1)	2490 (75.1)		103 (79.2)	309 (79.2)	
Consumer-driven^b^	258 (23.3)	774 (23.3)		26 (20.0)	78 (20.0)	
Comprehensive	17 (1.5)	51 (1.5)		1 (0.8)	3 (0.8)	
Comorbidities, n (%)						
Anemia	11 (1.0)	22 (0.7)	.364	1 (0.8)	3 (0.8)	1.000
Any atopic disorder^c^	252 (22.8)	577 (17.4)	<.001	32 (24.6)	65 (16.7)	0.059
Any autoimmune disorder^d^	55 (5.0)	72 (2.2)	<.001	8 (6.2)	10 (2.6)	0.097
Any cardiovascular disorder^e^	53 (4.8)	189 (5.7)	.285	9 (6.9)	28 (7.2)	1.000
Any mental health disorder^f^	143 (12.9)	539 (16.3)	.009	24 (18.5)	68 (17.4)	0.894
CCI score, mean ± SD	0.1±0.3	0.1±0.3	1.000	0.1±0.3	0.1±0.3	1.000

Abbreviations: AA, alopecia areata; AT, alopecia totalis; AU, alopecia universalis; CCI, Charlson Comorbidity Index.

^a^ Composite of health maintenance organization, preferred provider organization, point of service, and exclusive provider organization plans.

^b^ Composite of consumer-driven health plans and high-deductible health plans.

^c^ Composite of allergic rhinitis, asthma, atopic dermatitis, celiac disease, chronic urticaria, and conjunctivitis.

^d^ Composite of ankylosing spondylitis, Crohn’s disease, diabetes mellitus, Hashimoto’s disease, systematic lupus erythematosus, psoriasis, rheumatoid arthritis, Sjögren’s syndrome, ulcerative colitis, and vitiligo.

^e^ Composite of atherosclerosis, chest pain, dyspnea, heart palpitations, and shortness of breath.

^f^ Composite of attention deficit hyperactivity disorder, anxiety disorders, depression, obsessive-compulsive disorder, and substance abuse.

### HCRU and Medication Use

Relative to controls, patients with non-AT/AU had more PPPY outpatient (11.6 vs 8.0) and dermatologist (3.4 vs 0.4) visits (both *P<*.001) but similar rates of psychiatrist and psychologist visits during the follow-up period (Table 2). A higher difference was observed for patients with AT/AU vs controls in outpatient (14.5 vs 7.1) and dermatologist (3.6 vs 0.3) visits compared with the non-AT/AU sample (both *P<*.001). Moreover, a higher percentage of all patients with AA had at least 1 dermatologist visit, including both the non-AT/AU (81.7% vs 16.5%) and AT/AU (71.5% vs 14.4%) subgroups, vs controls during the follow-up period (all *P<*.001). Higher rates of corticosteroid use were observed during the study follow-up period in the non-AT/AU subgroup relative to matched controls. This included higher topical (36.4% vs 1.5%), injectable (51.4% vs 0.9%), and oral (11.0% vs 7.1%) corticosteroid use (all *P<*.001). Among patients with AT/AU, corticosteroid use was also higher vs controls, including for topical (29.2% vs 0.3%), injectable (29.2% vs 1.5%), and oral (28.5% vs 5.1%) corticosteroids (all *P<*.001). Rate of methotrexate use was significantly higher among adolescent patients with AA compared with matched controls, an increase that was more pronounced in the AT/AU subgroup vs matched controls (8.5% vs 0.3%) than in non-AT/AU vs matched controls (1.3% vs 0.1%) (all *P<*.001).

**Table 2.** HCRU and AA-related Medication Use During the Study Follow-up Period

	**Patients With Non-AT/AU**			**Patients With AT/AU**		
**Outcome**	**Non-AT/AU (n=1105)**	**Matched Controls Without AA (n=3315)**	***P* Value**	**AT/AU (n=130)**	**Matched Controls Without AA (n=390)**	***P* Value**
HCRU visits, mean ± SD						
Outpatient visits	11.6±11.6	8.0±12.8	<.001	14.5±11.8	7.1±9.6	<.001
Dermatologist visits	3.4±5.1	0.4±1.7	<.001	3.6±4.3	0.3±1.0	<.001
Psychiatrist visits	0.3±2.3	0.3±1.6	.559	0.1±0.7	0.3±1.3	.272
Psychologist visits	0.3±1.8	0.4±3.7	.278	0.2±1.6	0.1±0.7	.199
Any dermatologist visit, n (%)	903 (81.7)	548 (16.5)	<.001	93 (71.5)	56 (14.4)	<.001
Any psychiatrist visit, n (%)	55 (5.0)	195 (5.9)	.293	7 (5.4)	22 (5.6)	1.000
Any psychologist visit, n (%)	45 (4.1)	140 (4.2)	.896	6 (4.6)	14 (3.6)	.792
Medication use, n (%)						
Corticosteroids						
Topical	402 (36.4)	51 (1.5)	<.001	38 (29.2)	1 (0.3)	<.001
Injectable	568 (51.4)	30 (0.9)	<.001	38 (29.2)	6 (1.5)	<.001
Oral	121 (11.0)	234 (7.1)	<.001	37 (28.5)	20 (5.1)	<.001
Methotrexate	14 (1.3)	1 (0.1)	<.001	11 (8.5)	1 (0.3)	<.001
Mental health						
Antidepressants	82 (7.4)	312 (9.4)	.051	16 (12.3)	27 (6.9)	.081
Anxiolytics	48 (4.3)	129 (3.9)	.565	5 (3.8)	12 (3.1)	.887

Abbreviations: AA, alopecia areata; AT, alopecia totalis; AU, alopecia universalis; HCRU, healthcare resource utilization.

HCRU visits are summarized per patient per year.

### Payer and OOP Costs

**Figure 3.**
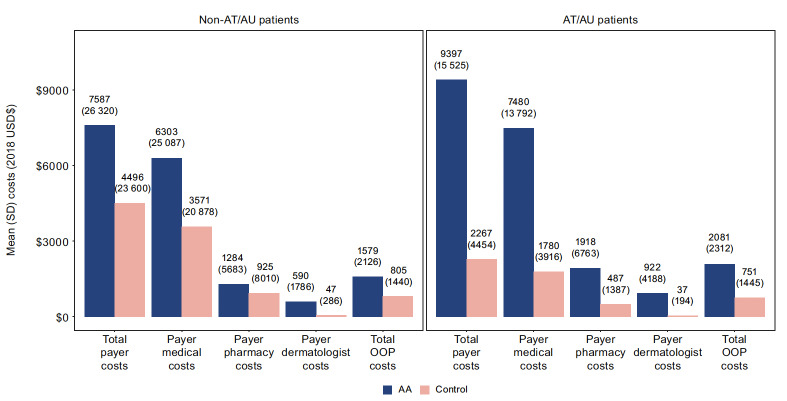
All-cause Payer and OOP Costs During the Study Follow-up Period Costs are summarized per patient per year. Payer medical and payer pharmacy costs add up to the total payer costs (except for AT/AU cohort, due to rounding). Payer dermatology costs are a subset of payer medical costs. OOP costs include enrollee payments made toward deductible, copays, and coinsurance. Values in parentheses (above the bars) are the SD. Abbreviations: AA, alopecia areata; AT, alopecia totalis; AU, alopecia universalis; OOP, out-of-pocket; USD$, US dollar.

Post-index, AA patients in the non-AT/AU subgroup had higher PPPY total payer costs compared with matched controls ($7587 vs $4496, *P<*.001) (Figure 3). Specifically, patients with non-AT/AU had higher medical ($6303 vs $3571, *P<*.001) and pharmacy ($1284 vs $925, *P=*.168) costs vs matched controls. PPPY OOP costs were similarly higher among the non-AT/AU subgroup compared with matched controls ($1579 vs $805, *P<*.001). Relative to the difference between non-AT/AU vs the matched-control subgroups, AT/AU had larger differences in total payer costs ($9397 vs $2267), including payer medical ($7480 vs $1780) and pharmacy ($1918 vs $487) costs, during the study follow-up period (all *P<*.001). The AT/AU subgroup also had relatively larger differences vs matched controls in OOP costs ($2081 vs $751, *P<*.001) compared with the non-AT/AU subgroup’s difference vs matched controls.

## DISCUSSION

This study assessed HCRU, costs, and medication use associated with adolescents with AA in a large cohort of commercially insured patients. To our knowledge, this is one of the first studies to explore treatments utilized in an adolescent population with AA in the United States. Our findings showcased that adolescent patients (AT/AU or non-AT/AU) incurred greater total direct costs than their respective matched-control groups, including higher medical and pharmacy costs and OOP costs. These results suggest that AA is not simply a “cosmetic” disease but instead a medical condition that can impose a substantial burden on both patients and health plans. The AT/AU subgroup is associated with the greatest mean total plan costs relative to controls. Similarly, adolescents with AA have more yearly outpatient and dermatologist visits compared with unaffected controls, and patients with AT/AU disease have higher HCRU. Corticosteroid use is also substantially higher in the AA group vs matched controls, including for topical, injectable, and oral treatments. Our analysis found that the incremental use of topical and injectable corticosteroids vs controls was higher among patients with non-AT/AU compared with AT/AU, whereas the incremental use of oral corticosteroids vs controls was higher among those with AT/AU. This finding reflects the higher rate of systemic treatment for patients with AT/AU who generally have a higher percentage of scalp involvement. Taken together, this research highlights the substantial burden of adolescent AA and provides new insights into the relative burden of AT/AU disease.

Our findings are consistent with and build on previous research that demonstrated a high economic burden of AA in a combined population of adolescents and adults.^41^ Similar to the present study, Xenakis et al^41^ observed significantly higher mean 12-month all-cause costs for patients with AA vs matched controls ($8853 vs $5548), which were driven by significant increases in medical ($4138 vs $2266) and pharmacy ($2422 vs $1372, all *P<*.0001) costs. The Xenakis et al study extends the present findings by similarly showing a greater difference in mean total costs among patients with AT/AU vs matched controls compared with non-AT/AU vs matched controls ($12,654 vs $8490, *P<*.0001) in a cohort of commercially insured adolescent patients.

The present findings also build upon a growing body of evidence evaluating the OOP cost burden of AA. To date, 2 survey studies have assessed the self-reported OOP costs and financial burden of AA among patients recruited from the National Alopecia Areata Foundation patient database.^40,42^ First, Li et al^42^ reported median overall OOP costs of $1354 annually among 675 adult participants, with most participants rating their financial burden of AA as moderately (31.7%) or seriously (25.2%) burdensome.^40,42^ In contrast to this prior study, the OOP costs of $1579 in patients with non-AT/AU and $2081 with AT/AU in the present study are focused on adolescents between the ages of 12 and 17 years and confirmed through insurance billing claims from a larger, comprehensive sample. Second, Mesinkovska et al^40^ analyzed a similar sample of 216 adult patients with AA and found high OOP expenditures for items such as wigs or hairpieces and psychotherapy visits, with a mean of approximately $2000 per year for each. The present study results affirm the high OOP costs associated with AA across a broad population and highlight the greater incremental OOP costs among adolescents with AT/AU compared with those without AT/AU.

In addition to showing greater cost and HCRU burden among patients with AT/AU, our findings demonstrated that these patients may have different patterns of medication use in a real-world setting than those with less extensive disease. Compared with non-AT/AU patients, those with AT/AU had substantially higher rates of oral corticosteroid use and lower rates of injectable corticosteroid use during the follow-up period. This pattern appears consistent with the latest recommendations from the Alopecia Areata Consensus of Experts study.^37^ Among this international panel of experts, intralesional corticosteroids were favored for adolescents and adults with limited disease, whereas topical or oral corticosteroids were preferred for patients with extensive disease.^37^ In particular, daily administration of oral prednisolone (or prednisone) was considered appropriate for patients with extensive AA.^37^ Further research is needed to improve understanding of the real-world treatment patterns among patients with AA and to inform novel therapeutic strategies.

These findings should be interpreted in the context of the study design. The data used in this study were representative of the commercially insured population in the United States. The patient sample in this study was also large, which contributed to a well-powered analysis and the ability to assess key subgroups of interest (ie, AT/AU). Furthermore, the matching procedure employed in this study allowed for balanced comparisons between cohorts, thereby reducing the risk of bias because of other variables. For example, the present study matched patients based on their baseline comorbidities (ie, CCI score), which is an important potential confounder that was not accounted for in prior research on the economic burden of AA.^41^

### Limitations

The present study was also subject to certain limitations. As a consequence of a lack of clinical information in administrative claims, patients with a diagnosis of AA could not be stratified by degree of hair loss aside from AT and AU manifestations. This limitation was addressed by identifying patients with an AT/AU diagnosis at any time, not just on the index claim, which is justified by the fact that AT/AU may not present the phenotype until later, and AT/AU codes may be underreported.^43^ Claims-based studies may be subject to incomplete, inaccurate, or missing data that could bias the results. This limitation was minimized by only including patients who had continuous health plan enrollment and at least 2 claims with a diagnosis of AA. Furthermore, the comparisons between AA and control cohorts may have been subject to unobserved or unmeasured confounders not included in the matching algorithm. Lack of payer coverage^44^ and available treatments may also lead these values to be underestimates of the true cost burden of AA.^40^ Similarly, the OOP costs in this study were based on insurance billing data and do not represent overall OOP costs patients may incur for AA treatment not covered. The increased burden observed among patients with AA may be attributable to AA itself or to associated comorbidities or underlying factors; more research is warranted to understand this attribution. Finally, although the data used were representative of the commercially insured US population, the study findings may not be generalizable beyond adolescents with commercial or Medicare supplemental insurance coverage, or those with other types of alopecia.

## CONCLUSIONS

This study provides real-world evidence in the commercially insured US population that AA is associated with a significant economic burden among one of the most vulnerable patient populations—adolescents—who are at a critical period of developing their self-identity.^45^ Adolescents with AA had significantly higher HCRU and all-cause costs from a payer and OOP perspective than their matched controls. This incremental economic burden was even greater among AA patients in the AT/AU subgroup when compared with the non-AT/AU subgroup. The present study is one of few assessing the economic burden of AA among adolescent patients in a real-world setting using evidence from US administrative claims data. The findings of this study document the effect and burden of this understudied autoimmune condition.

### Disclosures

M.R., K.G., and V.S. were employees of Pfizer and held stocks/stock options of Pfizer at the time of study conduct and analyses. E.S., C.C., T.W., N.D., and J.S. are employees of Analysis Group, Inc, a consultancy that received payment from Pfizer for participation in this analysis. A.M. reports consulting fees from AbbVie, Bioniz, Digital Diagnostics, Eli Lilly, hims™, and Pfizer; has received licensing/royalties from Concert and Pfizer; has served on the medical advisory board for hims™; has been an investigator in clinical trials for Concert and Eli Lilly; and is an associate editor of *JAMA Dermatology*.
